# Isotretinoin Concerns in Switzerland: A Student-Based Transversal Study

**DOI:** 10.3390/jcm14061801

**Published:** 2025-03-07

**Authors:** Anna-Lena U. Jakobi, Andreas J. Bircher, Alberto Pagnamenta, Isabella Terrani

**Affiliations:** 1 Faculty of Biomedical Sciences, Università della Svizzera Italiana, 6900 Lugano, Switzerland; andreas.bircher@usi.ch; 2 Clinical Trial Unit, Ente Ospedaliero Cantonale (EOC), 6900 Lugano, Switzerland; alberto.pagnamenta@eoc.ch; 3 Department of Intensive Care Medicine, Ente Ospedaliero Cantonale (EOC), 6900 Lugano, Switzerland; 4 Division of Pneumology, University of Geneva, 1211 Geneva, Switzerland; 5 Department of Dermatology, Ente Ospedaliero Cantonale (EOC), 6900 Lugano, Switzerland; isaterri@gmail.com

**Keywords:** isotretinoin, acne, non-adherence, phobia

## Abstract

**Background/Objectives**: Concerns about isotretinoin may affect both initiation and adherence in acne patients. We conducted a student-based transversal study including participants with knowledge on isotretinoin to assess related concerns and factors as well as the impact of an isotretinoin fact sheet in a before–after design. To our knowledge, no such surveys have been published to evaluate such concerns. **Method**: An online questionnaire about isotretinoin treatment was distributed by social media. The impact of written information about isotretinoin was assessed. **Results**: A total of 528 participants had fulfilled inclusion criteria. Most participants (53.8%) reported having concerns about isotretinoin treatment, mainly about xerosis cutis, but also about depression. A total of 49.1% of participants with a history of isotretinoin treatment reported that these concerns affected their adherence to treatment. Participants mostly relied on information from relatives or acquaintances and treating physicians, while reading a text about isotretinoin did not affect their concerns (*p* = 0.22). Multivariable regression analysis demonstrated that female participants, pharmacy students, and subjects with a history of acne had higher isotretinoin concerns. **Conclusions**: Students in Switzerland were concerned about an isotretinoin treatment. Written information did not change their existing concerns. This study highlights the importance for more education regarding potential side effects of isotretinoin.

## 1. Introduction

Acne represents a huge burden, especially for young people. It affects an estimated 9.4% of the world’s population, with the majority aged between 15 and 24 years [1]. In addition, acne is considered a major risk factor for depression and low self-esteem [2].

Oral isotretinoin has a high safety profile and is an effective treatment for severe refractory nodulocystic acne even in resistant cases [3]. Even low doses of oral isotretinoin have been reported to clear lesions in most treated patients [4].

Despite its high safety profile, isotretinoin therapy is associated with known side effects, including mucocutaneous manifestations such as cutaneous xerosis and pruritus, sensitivity to sun light, alterations in lipid and liver enzyme profiles, myalgia, and concerns regarding potential depression and teratogenicity [2,3,5,6,7]. These considerations have given rise to what we term “Isotretinoin Phobia” (IP)—a phenomenon similar to the well-documented corticosteroid phobia, where concerns about the medication impact treatment initiation and adherence [8,9].

Current research reveals gaps in understanding patient and parent concerns regarding isotretinoin. Recent studies highlight persistent misinformation, particularly on social media platforms, and ongoing patient apprehension despite updated safety data. Notably, no comprehensive epidemiological studies have evaluated population attitudes toward isotretinoin use independently of treatment history [10,11].

This study aims to determine the prevalence of isotretinoin-related concerns among mainly Swiss students, identify factors associated with IP, and assess whether enhanced knowledge about isotretinoin influences treatment concerns. These findings could provide valuable guidance for clinical practice.

## 2. Materials and Methods

### 2.1. Participant Population

The target population targeted mainly students of any gender in Switzerland who were connected via the social network of the authors.

### 2.2. Project Design

Student-based transversal study.

### 2.3. Data Collection

Data collection occurred between 10 December 2020 and 24 March 2021. An online questionnaire in English was designed using the “Zoho Survey” platform (https://www.zoho.com/de/survey/, accessed on 10 December 2020). The link to the questionnaire was mainly distributed to medical students and subsequently to their peers via social media platforms such as WhatsApp, Instagram, and Facebook.

### 2.4. Questionnaire

**Figure 1:**
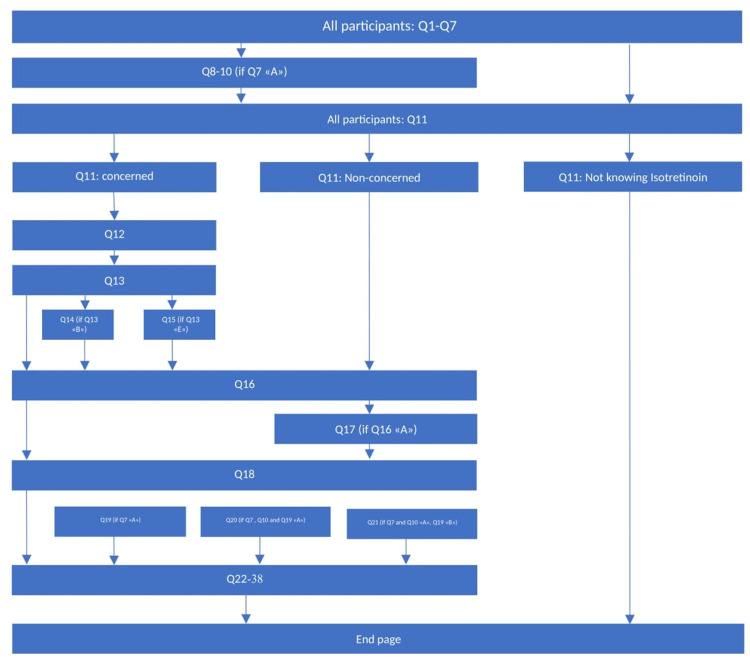
Questionnaire structure—depending on the answers given, not all 37 questions appeared to every participant. The arrows show which questions followed and, in brackets, what had to be fulfilled for the specific question to pop up.

The questionnaire (Appendix A), adapted from an existing study on corticosteroid concerns [9], comprised 37 questions across four sections: demographics, acne-related health status and treatment experiences, isotretinoin knowledge assessment, isotretinoin concerns before and after reading a text about isotretinoin, and the Hospital Anxiety and Depression Scale (HADS), a standardised 14-item self-report validated questionnaire that measures depression and anxiety [12]. Based on their response to isotretinoin concerns Q11 (“Irrespectively if you have acne or not, from what you currently know, do you have any concerns related to a treatment with isotretinoin (Roaccutan^®^, Curakne^®^, Tretinac^®^, …)?”), participants were categorized into three groups (Figure 1):

- Isotretinoin-Phobic Participants (IPPs)—with concerns about isotretinoin therapy;
- Non-Isotretinoin-Phobic Participants (NIPPs)—no concerns regarding isotretinoin therapy;
- Participants with no prior knowledge of oral isotretinoin.

The latter group was excluded from subsequent statistical analyses and received a closing statement.

### 2.5. Main Study Population

Participants who answered Q11 with either “yes” or “no” were included as Isotretinoin-Phobic Participant/s (IPP) and Non-Isotretinoin-Phobic Participant/s (NIPP), respectively. Participants who did not complete the full questionnaire beyond Q11 did not drop out. Instead, pairwise deletion (available-case analysis) was used rather than multiple imputation.

Participants who either did not complete all questions Q1–10 or participants with no knowledge of oral isotretinoin were excluded from the statistical analyses.

### 2.6. Statistical Analysis

#### 2.6.1. Reliability of Questionnaire

We adapted the original questionnaire on topical corticosteroid concerns for our study and therefore assessed internal consistency of the questionnaire by calculating the Cronbach’s alpha [13].

#### 2.6.2. Sample Size Estimation

Sample size estimation, accounting for unknown prevalence of isotretinoin concerns (10–90% range), indicated a minimum requirement of 385 participants. A prevalence below 10% impacted the sample size: for instance, an expected prevalence of 2% would have required a sample size of 753 [14].

#### 2.6.3. Data Analysis

Quantitative variables were summarised as the mean with standard deviation (SD) or as the median with the 25th and the 75th percentiles, where appropriate. Comparisons of the various random variables of interest between IPPs and NIPPs were made using parametric (Student *t*-test) and non-parametric (Mann–Whitney or chi-squared test, as appropriate) test statistics. We then performed a multivariable logistic regression analysis to identify potential demographic predictors of isotretinoin concerns. Unadjusted and adjusted odds ratios (ORs) were calculated and expressed with the corresponding 95% confidence intervals (95%-CI). The effects on concern of reading a text about isotretinoin were assessed with the Wilcoxon-rank test. All tests were performed two-sided, and a value of *p* < 0.05 was considered statistically significant. All analyses were performed using Stata 15 (StataCorp LP, College Station, TX, USA).

## 3. Results

Internal consistencies assessed by Cronbach’s alpha for all items were between 0.957 and 0.963, indicating a high reliability of the questionnaire.

**Figure 2:**
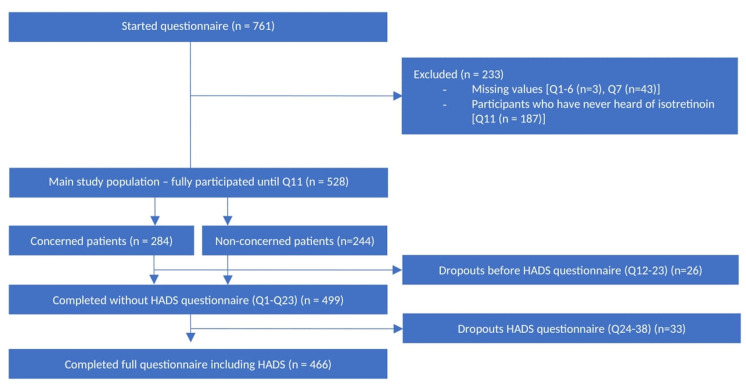
Flow chart exclusions and dropouts.

A total of 761 subjects started compiling the questionnaire, and 528 participants were included for statistical analysis (main study population) of whom 88% (466 participants) completed the entire questionnaire (Figure 2). The main study population (Table 1) had a mean age of 24.7 years (95% confidence interval: 24.1–25.2 years), was predominantly female (77.0%), had at least a college or bachelor’s degree (81.7%), and was mainly studying medicine (51.6%).

The majority (n = 284; 53.8%) expressed concerns related to treatment with isotretinoin (Figure 2).

Participants with no previous knowledge of isotretinoin were not included in the subsequent statistical analyses and received a closing statement.

The univariate analysis (Table 1) revealed two predictors for a higher isotretinoin concern (pharmacy students and female participants). This finding was validated in the multivariable logistic regression analysis (Table 2), and moreover, a history of acne was found to be associated with heightened concerns.

Other factors such as age, ethnicity, education level, studying medicine, and previous treatment with isotretinoin showed no significant associations (*p* > 0.05).

Xerosis cutis and mucous membranes were among the top concerns of IPPs and most of IPPs’ knowledge and concerns about isotretinoin came from relatives or acquaintances (Table 3).

**Figure 3:**
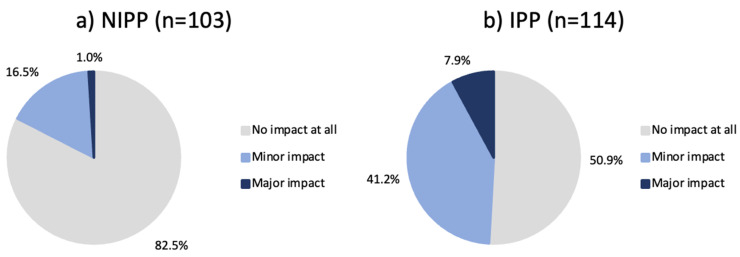
(**a**) NIPPs and (**b**) IPPs with a history of isotretinoin treatment and their self-reported impact of concerns on adherence to isotretinoin treatment. NIPP: Non-Isotretinoin-Phobic Participant/s, IPP: Isotretinoin-Phobic Participant/s.

Participants who reported that “school/university” had the largest impact on their concerns were mostly concerned (n = 76; 66.7% IPPs), whereas most participants who reported that dermatologists and family doctors had the largest impact on their knowledge had no concerns (n = 122; 55% NIPPs). Among those treated with isotretinoin, 17.5% of NIPPs and 49.1% of IPPs reported at least a minor impact on the therapy (Figure 3).

**Figure 4:**
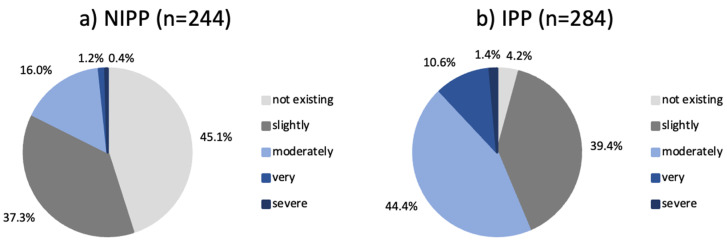
Main study population (**a**) NIPPs and (**b**) IPPs and their self-reported grading of concerns. NIPP: Non-Isotretinoin-Phobic Participant/s, IPP: Isotretinoin-Phobic Participant/s.

When asked to quantify their concerns, the majority (n = 406; 76.9%) of participants expressed at least slight concern regarding isotretinoin therapy. A discrepancy was observed in the levels of concern exhibited by IPPs and NIPPs. The vast majority of IPPs (95.8%) assessed their concerns as at a minimum “slight”, and still 54.9% of NIPPs expressed at least a slight degree of concern. The difference in “no concern” between the two groups was tenfold, namely 4.2% for the IPPs and 45.1% for the NIPPs (Figure 4).

Reading an information leaflet did not meaningfully impact isotretinoin concern. Only a minority (28.5%) had their concerns changed after reading an information leaflet about isotretinoin. The most frequently selected reasons for changing were information about mood changes, depression and increased risk of suicide, changes in laboratory parameters, and long-term clinical experience with isotretinoin.

Most participants completed the 14-item HADS questionnaire (Figure 2). For depression, the median score was 2 points, with 25th and 75th percentiles at 1 and 5 points, respectively, for both NIPPs and IPPs. For anxiety, the median HADS score was 6 points for IPPs (25th percentile 3 points; 75th percentile 9 points) and 5 points for NIPPs (25th percentile 3 points, 75th percentile 8 points). Both scores were below the borderline threshold of 8 points [15], and there was no statistically significant difference between IPPs and NIPPs for both depression and anxiety.

## 4. Discussion

The aim of this study was to investigate, in a non-clinical setting, whether students living in Switzerland have concerns about isotretinoin treatment and to assess the characteristics in relation to their concerns about isotretinoin.

This study revealed that the majority of the study population exhibited relevant concerns regarding an isotretinoin-based acne treatment. Factors such as educational background and prior use of isotretinoin did not demonstrate a significant impact on these concerns. Receiving written information about isotretinoin was unlikely to change a participant’s opinion by itself.

We observed that female participants were more worried than male participants (Table 1). In a study on “Steroid Phobia” by Contento et al. [16], female gender similarly tended to be associated with greater phobia of the medication. Kessler et al. [17] found in their study on the lifetime prevalence of DSM-IV disorders that women had a significantly higher risk for affective and anxiety disorders while another study on pharmacophobia in general did not identify female gender as a predictor [18]. Further studies are needed to confirm the observed the correlation between gender and concerns about isotretinoin.

In addition, pharmacy students expressed significantly higher levels of concern than the general study population, as well as significantly higher levels than medical students (Table 1 and Table 2). To the best of our knowledge, there is an absence of studies that have examined the differential levels of fear regarding medication between medical and pharmacy students.

Furthermore, our findings indicate that participants who primarily sought information from dermatologists and family doctors exhibited a reduced level of concern in comparison to those who predominantly relied on information from school or university (Table 3). While there is an absence of comparable studies on medication information sources, there is ample evidence to suggest that effective counselling by the treating physician can aid in reducing patients’ phobias towards medications. For instance, a Korean study on Topical Corticosteroid (TCS) phobia in children with atopic eczema demonstrated that a 10- to 15-min session conducted by a dermatologist, in conjunction with written instructions, resulted in a reduction of over 40% in the phobia index score [19].

Moreover, the results of the present study (Table 3) highlight the impact of family and friends on IPPs. The results of this study demonstrate a congruence with those obtained in previous research on TCS phobia [8,16,20]. The findings indicate that negative reports from family members and friends have a detrimental influence on the level of concern experienced by the individual. A Turkish study with parents of adolescent acne patients suggests that limited knowledge about isotretinoin treatment among parents can negatively affect the adolescent’s perception of a medication [21]. This research provides evidence in support of the incorporation of family members for the education on isotretinoin treatment. In a similar manner to the present study, a study on the management of irritable bowel syndrome has shown that younger and mid-aged adolescents benefit from family support and are more likely to adhere to their prescribed medication [22].

Furthermore, a study investigating the nature of content posted on the social media platform Instagram with respect to the systemic acne medication isotretinoin and the use as a potential surveillance tool for monitoring adverse effects associated with isotretinoin treatment found that 41% of Instagram posts assessed referenced adverse effects of oral isotretinoin, predominantly dry facial skin or cracked lips. The study concluded that social media could serve as a valuable surveillance tool for monitoring the general burden of adverse effects. In connection with our study, the study demonstrated that individuals express concerns and discuss the topic on social media, disseminating their opinions through this medium [23].

While acne status was not associated with isotretinoin concerns in the univariate analysis, concerns emerged in the multivariate logistic regression model (OR = 1.555, 95% CI [1.028, 2.353], *p* = 0.037) when controlling for potential confounding variables such as gender and study discipline. A comparable study on the utilisation of topical corticosteroids yielded analogous outcomes, with individuals afflicted by the condition being treated exhibiting heightened concerns [20].

Half of the IPP population reported that their concerns had at least a minor impact of concerns on their adherence (Figure 3). Research conducted about TCS phobia has shown analogous results with respect to the non-adherence of patients with concerns, suggesting that further research into counselling to prevent or reduce side effects may be of interest to improve adherence to isotretinoin treatment [8,19,20,24].

The provision of educational resources on how to manage side effects could be a viable option to increase compliance. For instance, in the present study, xerosis cutis was identified as the most feared side effect, and it was found to be amenable to education on the correct application of emollients [25]. In cases where pruritus is a consequence, the use of antihistamines as an adjunctive measure when emollients are ineffective is recommended [26]. Whilst isotretinoin has been shown to induce skin and mucosa dryness, resulting in pruritus, it is also employed in the treatment of highly pruritic conditions, such as lichen planus, pityriasis rubra pilaris, ichthyosis, and chronic pruritus of unknown origin [27,28]. Further investigation is required to elucidate the relationship between pruritus and retinoids.

Educational initiatives were recommended to prevent the worsening of cutaneous sensitivity, and the use of sun protection measures was advised to mitigate the adverse effects of ultraviolet radiation, which could otherwise lead to a worsening of skin condition and an increase in associated adverse effects [25,26].

Also, it would be advisable for physicians to educate their patients more comprehensively on foetal complications and proactively offer to discuss the washout period with them. This would serve to eliminate or lessen any concerns regarding long-term teratogenic risks [29]. Healthcare providers can provide patients with a greater level of reassurance and should implement preventative measures to manage side effects. Communicating the outcomes of treatment, such as clearer skin and less scarring, and the subsequent reduction in acne-related distress, has great potential to outweigh concerns about the predominantly transient nature of side effects. It is important to note that dosage adjustments of isotretinoin can help to mitigate side effects since many of the side effects of the drug are dose dependent [4]. Whether the long-term benefits of the treatment or the short-term management of side effects should be prioritised in the treatment approach merits further discussion and research.

The majority of participants reported that their opinion of isotretinoin remained unchanged subsequent to reading an information leaflet. This finding aligns with the findings of studies conducted on TCS phobia, which indicated that impersonal interventions such as educational videos, standardised written information, or online forums were ineffective in reducing the prevalence of TCS phobia. Conversely, the literature suggests that fostering a positive doctor–patient relationship, coupled with education about TCS, can be an effective strategy for reducing phobia [8,16,19].

Despite the fact that the information leaflet did not result in a notable shift in opinion regarding isotretinoin, 28.5% of the population indicated that they had modified their assessment in light of the information provided on depression, mood changes, an elevated risk of suicide, alterations in laboratory parameters, and long-term clinical experience with isotretinoin. A subgroup analysis was not prespecified in the research protocol, and to avoid the creation of false positive findings, the exacerbation or mitigation of concerns was not addressed. Nevertheless, the results underscore the necessity for further education regarding potential side effects of isotretinoin, such as depression. As indicated by another study, patients undergoing isotretinoin treatment exhibited even lower levels of depressive symptoms after therapy compared to those not undergoing any intervention, largely attributed to the psychological burden of acne [30]. Future research should explore the efficacy of different educational interventions beyond written information.

The present study’s population was not biased by varying levels, and no increased rates of depression or anxiety were observed, as determined by the HADS questionnaire, which is frequently used in studies to evaluate depression and anxiety levels before and after acne treatment [31,32].

This study is subject to several limitations. Firstly, this study was based on a self-reported survey, with participants selected through the authors’ social media network. This resulted in a study population consisting mostly of well-educated female medical students. No measures were implemented to mitigate selection bias.

Secondly, non-students were not excluded from this study as the questionnaire did not specifically ask participants whether they were currently studying. Rather, it asked them to specify the field in which they were studying/working. As the questionnaire was mainly distributed by the co-authors to students in a university setting, it is assumed that non-students also represent a limited number of subjects.

Thirdly, the name of the online questionnaire, “Isotretinoin Phobia”, might have led to a higher number of IPPs due to the negative impact of the word “phobia” on perception.

Finally, the completion of the questionnaires occurred during the period of the pandemic of the Coronavirus. Despite the unbiased nature of the study population with regard to depression and anxiety, other studies have indicated that anxiety and depression were more prevalent during this period [31,32,33].

## 5. Conclusions

In conclusion, the present study suggests that a considerable number of Swiss students have concerns regarding isotretinoin treatment. Except for gender, an educational background in pharmacy studies, and acne status, demographics did not appear to have a significant impact on the level of fear experienced. The perception of isotretinoin was found to be influenced by family members, acquaintances, and physicians rather than other sources. Consequently, information campaigns on isotretinoin should consider targeting the acne patient environment and include information on relevant topics such as the prevention of the most commonly concerning side effects, such as dry skin and mucous membranes.

## Figures and Tables

**Table 1:** Participant characteristics (variables) associated with Isotretinoin-Phobic Participant/s (IPP) and Non-Isotretinoin-Phobic Participant/s (NIPP) in the univariate analysis; odds ratio (OR), 95% Confidence Interval (CI).

Variable	NIPPs, n (%), Mean ± SD	IPPs, n (%), Mean ± SD	*p*-Value
Age	24.5 ± 5.7	24.8 ± 7.4	0.652
Female sex	175 (72.02)	229 (81.21)	0.044
Ethnicity			0.536
European	235 (96.71)	275 (97.17)	0.95
Education			0.685
Secondary school	3 (1.24)	2 (0.71)	
Apprenticeship/vocational training	8 (3.31)	10 (3.55)	
High school	34 (14.05)	39 (13.83)	
College or bachelor’s degree	127 (52.48)	134 (47.52)	
Master’s degree or higher	70 (28.93)	97 (34.40)	
Study/working discipline			
Pharmacy	23 (9.43)	57 (20.07)	0.004
Medicine	131 (53.69)	140 (49.296)	0.6
Others	90 (36.89)	87 (30.63)	0.297
History of acne	175 (71.72)	219 (77.11)	0.156
History of isotretinoin treatment in participants with a history of acne	103 (58.86)	116 (52.97)	0.243

**Table 2:** Potential predictor variables associated with isotretinoin concern. Multivariable logistic regression analysis with Isotretinoin-Phobic Participant/s (IPP) and Non-Isotretinoin-Phobic Participant/s (NIPP); odds ratio (OR), 95% Confidence Interval (95%-CI).

Variable	OR	95% CI	*p*-Value
Age (per year)	1.016	0.988–1.045	0.286
Sex (female as reference)	0.601	0.399–0.905	0.015
Ethnicity (Caucasian as reference)	0.824	0.498–1.365	0.452
Education (college as reference)	1.041	0.832–1.303	0.724
Study/working discipline (other than pharmacy and medicine as reference)			
Pharmacy	2.708	1.496–4.902	0.001
Medicine	1.102	0.748–1.625	0.623
Acne status (never affected as reference)	1.555	1.028–2.353	0.037

**Table 3:** (**a**) Specific concerns and (**b**) self-reported largest impact on knowledge/concerns in isotretinoin-concerned population. IPP: Isotretinoin-Phobic Participant/s.

(**a**) Specific Concerns in Isotretinoin-Concerned Population, IPPs (284/528), n (%)	
Dry skin or mucous membranes	196 (69.0)
Sensitivity to sun light	180 (63.4)
Fetal malformation	166 (58.5)
Abortion	166 (58.5)
Fertility problems	166 (58.5)
Liver disorder	119 (41.9)
Risk of depression or suicide	114 (40.1)
Increase in lipid profile (e.g., hypercholesterinaemia)	49 (17.3)
Worsening of acne lesions	39 (13.7)
Myalgia (muscle aches and pain)	35 (12.3)
Other (please specify)	8 (2.8)
(**b**) Self-reported largest impact on knowledge/concerns in isotretinoin-concerned population, IPPs (284/528), n (%)	
Advised by relatives or acquaintances (colleagues, friends)	114 (40.1)
Informed by a physician (family doctor, dermatologist, other)	111 (39.1)
Informed by school/university (lectures)	98 (34.5)
Informed upon this topic by the internet, TV, radio, social media, press article, medical journals	73 (25.7)
Personal bad experience with this treatment	65 (22.9)
Informed by a pharmacist	36 (12.7)

## Data Availability

All relevant data are within the manuscript. The data that support the findings of this study are available from the corresponding author upon reasonable request.

## Abbreviations

The following abbreviations are used in this manuscript:

HADS	Hospital Anxiety and Depression Scale
ICS	Inhaled Corticosteroids
IP	Isotretinoin Phobia
IPP	Isotretinoin-Phobic Participant/s
NIPP	Non-Isotretinoin-Phobic Participant/s
TCS	Topical Corticosteroid/s

## Appendix A

### Questionnaire

**Isotretinoin Phobia** This questionnaire wants to explore the level of knowledge, awareness, and the corresponding fears from acne therapy, in particular a treatment with oral „isotretinoin” (e.g., Roaccutan^®^, Curakne^®^, Tretinac^®^, …). Isotretinoin (a vitamin A derivate) is commonly used in acne therapy, usually in form of a pill over several months, and has been shown to be very effective for its treatment.	
**Part A) Demography**	
**Questions**	**Answers**
1. What is your age?	*Enter age*
2. What was your assigned sex at birth?	▪ Male ▪ Female ▪ Undetermined
3. What gender do you currently live as in your day-to-day life?	▪ Male ▪ Female ▪ Other
4. Please specify your ethnicity.	▪ European ▪ American ▪ Asian ▪ African ▪ Australian ▪ Other ▪ No answer
5. What is your highest level of education you have completed–or you intend to complete over the next 24 months?	▪ Compulsory school (“Primarschule”) ▪ Secondary school (“Realschule” or “Sekundarschule”) ▪ Apprenticeship/vocational training (Berufslehre) ▪ High school (Kantonsschule) ▪ College (Fachhochschule) or Bachelor’s degree ▪ Master’s degree or higher ▪ No answer
6. In what kind of discipline are you currently studying/working?	▪ Student (high school) ▪ Pharmacy ▪ Medicine ▪ Science and technology ▪ Economics ▪ Law and public policy ▪ Informatics ▪ Architecture and engineering ▪ Arts and culture ▪ Communications ▪ Community and social services ▪ Education ▪ Installation, repair and maintenance ▪ Other (please specify):
**Part B) Acne status** Acne is a skin disease that affects nearly 90% of people in western societies during their teenage years but can occur before adolescence and may persist into adulthood. Typical features of the condition include blackheads, whiteheads, pimples, oily skin, and possible scarring of the face, the upper part of the chest region, and back.	
**Question**	**Answers**
7. Have you ever been affected by acne?	- Yes (If yes, move to Q8) - No (If no, move to Q11)
**Part C) Experience with acne therapy**	
**Question**	**Answers**
8. What have been the acne treatment approaches over time? (Multiple answers possible)	▪ no therapy ▪ self-treatment ▪ guided by pharmacist ▪ guided by family doctor ▪ guided by dermatologist ▪ Other (please specify):
9. Have you undergone complementary therapies? (Multiple answers possible)	▪ Acupuncture ▪ Osteopathy ▪ Homeopathy ▪ No ▪ Other (please specify):
10. Did you ever receive a prescription for an oral isotretinoin (Roaccutan^®^, Curakne^®^, Tretinac^®^, …) therapy?	- Yes - No
**Part D) Existing concerns acne therapy**	
**Question**	**Answers**
11. Irrespectively if you have acne or not, from what you currently know, do you have any concerns related to a treatment with isotretinoin (Roaccutan^®^, Curakne^®^, Tretinac^®^, …)?	▪ Yes (go to Q12) ▪ No (go to Q16) ▪ I have never heard of this medicament before (last page → exclusion)
12. What are your specific concerns? (Multiple answers possible)	▪ Dry skin or mucous membranes ▪ Worsening of acne lesions ▪ Fetal malformation, abortion, fertility problems ▪ Liver disorder ▪ Increase in lipid profile (f.e. hypercholesterinemia) ▪ Myalgia (muscle aches and pain) ▪ Risk of depression or suicide ▪ Sensitivity to sun light ▪ Other (please specify):
13. Where do your concerns come from? (Multiple answers possible)	- Personal bad experience with this treatment - Informed upon this topic by the internet, TV, radio, social media, press article, medical journals (if B, go to Q14) - Advised by relatives or acquaintances (colleagues, friends) - Informed by a pharmacist - Informed by a physician (family doctor, dermatologist, other) (if E, go to Q15) - Informed by school/university (lectures)
14. What platform exactly were you informed by? (multiple answers possible)	▪ Internet ▪ TV ▪ Radio ▪ Social media ▪ Press article ▪ Medical journals
15. What kind of physician were you informed by?	▪ Family doctor ▪ Dermatologist ▪ Other (please specify):
16. Which source of information is currently for you the one which has the largest influence/impact on your knowledge on acne therapy with isotretinoin (Roaccutan^®^, Curakne^®^, Tretinac^®^, …)?	- Media - Relatives or acquaintances (colleagues, friends) - Pharmacist - Family doctor - Dermatologist - School/university (lectures) - Other (please specify):
17. What kind of media has the largest impact on your knowledge?	▪ Internet ▪ TV ▪ Radio ▪ Social Media ▪ Press article ▪ Medical journals
18. Please rate your doubts about isotretinoin therapy?	▪ Not concerned ▪ Slightly concerned ▪ Moderately concerned ▪ Very concerned ▪ Extremely concerned ▪ (If Q7“No” → go to part E)
19. Have you ever been treated with an oral isotretinoin?	- Yes (go to Q20) - No (go to Q21)
20. If you have been treated with an oral isotretinoin therapy, how much have your concerns had an impact on your therapy (shorter treatment duration, skipped medication)?	▪ My concerns had no impact at all. ▪ My concerns had a minor impact on the therapy. ▪ My concerns had a major impact on the therapy duration. ▪ (Go to Q22)
21. If you have NOT been treated with an oral isotretinoin therapy, how much have/had your concerns have/had an impact on your decision NOT to take isotretinoin?	▪ My concerns have/had no impact at all on the therapy decision. ▪ My concerns have/had a minor impact on the therapy decision. ▪ My concerns have/had a major impact on the therapy.
**Part E) Information on acne therapy with isotretinoin**	
**Question**	**Answers**
Please read the information below. - Isotretinoin is a derivative of vitamin A that has been available orally in Switzerland since 1983. Consequently, there are more than 25 years of experience with the side effects and benefits of the treatment. - To date, there are no doubts about its benefits: Oral Isotretinoin is the most enduring and effective treatment for nodulocystic acne and the best scar prevention. Isotretinoin acts on the keratinization of hair follicle cells, so that sebum can drain freely, reduces the size of the sebaceous glands and also has an anti-inflammatory property. - The most common side effect is dry skin respectively dry mucous membranes, which can be treated well with emollients or eye drops. - An initial worsening of acne is possible, but this is a temporary phenomenon. - Isotretinoin is highly teratogenic and the use of this drug is absolutely contraindicated during pregnancy or lactation. A safe contraception method is mandatory. - Other possible side effects include increased liver parameters, increased lipid profile, or myalgia. These side effects are regularly checked for and are reversible upon stopping the treatment - Several studies have shown that acne is associated with mood changes, depression and a higher risk of suicide. It is still controversial whether Isotretinoin slightly increases or even reduces these associated conditions. - Isotretinoin increases sensitivity to sun light; therefore, it is important that you use sunscreens.	
22. After reading this information, how strong are your doubts related to an Acne therapy with Isotretinoin now?	▪ Not concerned ▪ Slightly concerned ▪ Moderately concerned ▪ Very concerned ▪ Extremely concerned
23. If your assessment has changed, indicate which statement in the paragraph 19 above made you most change your mind	▪ A ▪ B ▪ C ▪ D ▪ E ▪ F ▪ G ▪ H ▪ My assessment has not changed
**Part F) HADS questionnaire**	
24. I feel tense or ‘wound up’:	a) Most of the time b) A lot of the time c) From time to time, occasionally d) Not at all
25. I still enjoy the things I used to enjoy:	a) Definitely as much b) Not quite so much c) Only a little d) Hardly at all
26. I get a sort of frightened feeling as if something awful is about to happen:	a) Very definitely and quite badly b) Yes, but not too badly c) A little, but it doesn’t worry me d) Not at all
27. I can laugh and see the funny side of things:	a) As much as I always could b) Not quite so much now c) Definitely not so much now d) Not at all
28. Worrying thoughts go through my mind:	a) A great deal of the time b) A lot of the time c) From time to time, but not too often d) Only occasionally
29. I feel cheerful:	a) Not at all b) Not often c) Sometimes d) Most of the time
30. I can sit at ease and feel relaxed:	a) Definitely b) Usually c) Not Often d) Not at all
31. I feel as if I am slowed down:	a) Nearly all the time b) Very often c) Sometimes d) Not at all
32. I get a sort of frightened feeling like ‘butterflies’ in the stomach:	a) Not at all b) Occasionally c) Quite often d) Very often
33. I have lost interest in my appearance:	a) Definitely b) I don’t take as much care as I should c) I may not take quite as much care d) I take just as much care as ever
34. I feel restless as I have to be on the move:	a) Very much indeed b) Quite a lot c) Not very much d) Not at all
35. I look forward with enjoyment to things:	a) As much as I ever did b) Rather less than I used to c) Definitely less than I used to d) Hardly at all
36. I get sudden feelings of panic:	a) Very often indeed b) Quite often c) Not very often d) Not at all
37. I can enjoy a good book or radio or TV program:	a) Often b) Sometimes c) Not often d) Very seldom
